# DNA Methylation mediated down-regulating of MicroRNA-33b and its role in gastric cancer

**DOI:** 10.1038/srep18824

**Published:** 2016-01-05

**Authors:** Haixin Yin, Peng Song, Rui Su, Guihua Yang, Lei Dong, Min Luo, Bin Wang, Bei Gong, Changzheng Liu, Wei Song, Fang Wang, Yanni Ma, Junwu Zhang, Weibin Wang, Jia Yu

**Affiliations:** 1 Department of Biochemistry, Institute of Basic Medical Sciences, Chinese Academy of Medical Sciences (CAMS) & Peking Union Medical College (PUMC), Beijing 100005, PR China; 2 Department of Surgery, The Shanxi Academy of Medical Sciences & Shanxi Dayi Hospital, Taiyuan 030032, PR China; 3 The First Surgery Department of Nanlou, Chinese People’s Liberation Army General Hospital, Beijing 100853, PR China; 4 Department of Clinical Laboratory, Beijing Shijitan Hospital, Beijing100038, PR China; 5 Department of General Surgery, Peking Union Medical College Hospital, CAMS & PUMC, Beijing 100005, PR China

## Abstract

The discovery of microRNAs (miRNAs) provides a new and powerful tool for studying the mechanism, diagnosis and treatment of human cancers. Currently, down-regulation of tumor suppressive miRNAs by CpG island hypermethylation is emerging as a common hallmark of cancer. Here, we reported that the down-regulation of miR-33b was associated with pM stage of gastric cancer (GC) patients. Ectopic expression of miR-33b in HGC-27 and MGC-803 cells inhibited cell proliferation, migration and invasion, which might be due to miR-33b targeting oncogene c-Myc. Moreover, enhanced methylation level of the CpG island upstream of miR-33b in GC patients with down-regulated miR-33b was confirmed by methylation-specific PCR (MSP) amplification. Furthermore, re-introduction of miR-33b significantly suppressed tumorigenesis of GC cells in the nude mice. In conclusion, miR-33b acts as a tumor suppressor and hypermethylation of the CpG island upstream of miR-33b is responsible for its down-regulation in gastric cancer.

Gastric cancer (GC) was once the second most common cancer worldwide and still the second cause of cancer-related deaths nowadays^1^. Throughout the last few decades it has become obvious that GC results from a variety of internal mechanism disorder and environmental factors^2^,^3^,^4^. So far, the early stage of GC development is often asymptomatic despite the advance in diagnosis and treatment. Although extensive studies have been performed to identify genetic pathways and genes involved in GC, the prognosis for patients remains poor and little improvement of long-term survival has been achieved. Therefore, looking for an effective tumor marker for early diagnosis or tumor prognosis of GC is extremely important.

MicroRNAs (miRNAs), a class of small non-coding RNAs, have been considered as critical regulators of gene expression. Numerous studies have showed that they play crucial roles in various types of human diseases, especially in tumorigenesis^5^. Although high-throughput microarray and/or RNA sequencing technology produced lots of miRNAs with aberrant expression in GC, only a few miRNAs were identified to correlate with gastric carcinogenesis. MiR-33b is one member of miR-33 family and locates at the intronic region of SREBF-1 gene encoding sterol regulatory element binding protein. SREBF-1 belongs to the basic-helix-loop-helix leucine zipper class of transcription factor and has been reported to regulate the synthesis of enzymes involved in sterol biosynthesis and affect both cell proliferation and cell cycle progression^6^. Najafi-Shoushtari *et al.* confirmed that inhibition of miR-33b could increase plasma HDL level of fatty acid oxidation, suggesting its potential therapeutic significance in dyslipidemia and metabolic diseases^7^. SachiInukai *et al.* reported that miR-33 family could not only regulate cholesterol and fatty acid metabolism together with their host genes SREBF, but also affect cell cycle and cell proliferation by suppressing cyclin-dependent kinase 6 (CDK6) and cyclin D1 (CCND1) to prevent cell proliferation and cell cycle progression^8^. Several studies reported abnormal expression of miR-33b in human diseases. For example, Abigail *et al.* found that miR-33b was up-regulated in human papilloma virus (HPV) -positive cases of squamous cell carcinoma of the head and neck (SCCHN) compared to the cases lacking HPV^9^. Ze *et al.* indicated the down-regulation of miR-33b in multiple myeloma and its regulatory roles in tumor cell proliferation, migration, and apoptosis^10^. Two previous publication, Lv SQ *et al.* and Apana *et al.* demonstrated that miR-33b acted as a tumor suppressor through targeting c-Myc in medulloblastoma^11^,^12^. However, it is not clear about the expression and roles of miR-33b in GC.

In this study, we assessed the expression level of miR-33b in clinical collection of 150 pairs of GC samples, and found that the down-regulation of miR-33b was associated with pM stage of GC patients. Ectopic expression of miR-33b inhibited cell proliferation, migration and invasion of GC cells, HGC-27 and MGC-803. Further investigation indicated that the tumor suppressive roles of miR-33b might be through directly repressing c-Myc. Moreover, re-introduction of miR-33b significantly suppressed gastric tumorigenesis *in vivo*. Since DNA methylation of gene regulatory region is a major mechanism that affects the expression of a subset of tumor suppressive miRNAs in human cancer, we subsequently evaluated the methylation state of three CpG islands upstream of miR-33b gene locus. The enhanced methylation level of one CpG island was in accordance with down-regulation of miR-33b in GC samples, suggesting that the down-regulation of miR-33b is, at least, partly induced by DNA methylation in gastric cancer.

## Results

### Expression of miR-33b in GC and its relationship with clinicopathological

#### factors

**Figure 1:**
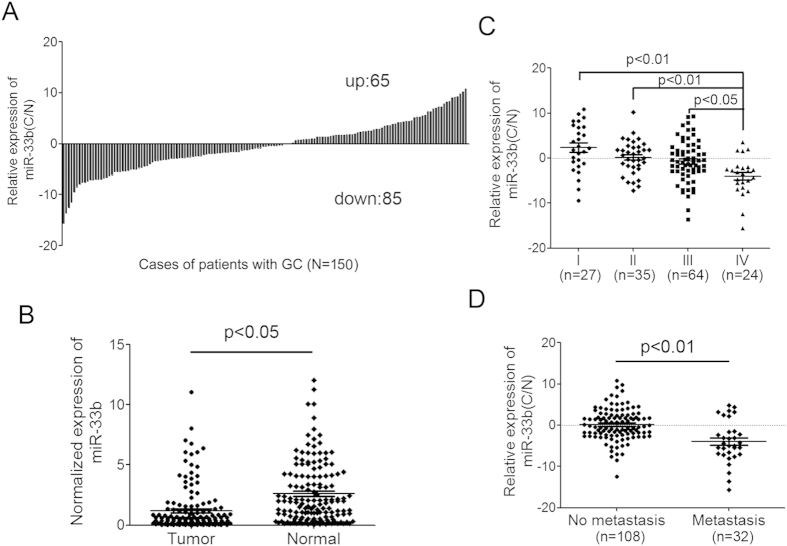
The expression analysis of miR-33b in GC tissues and its relationship with clinicopathological factors. (**A**) miR-33b was detected in 150 GC patients by q-RT-PCR. Data was presented as log 1.5 of fold change of GC tissues relative to adjacent normal regions. (**B**) Relative miR-33b expression levels in primary GC tissues and adjacent normal regions. (**C**) The down-regulated miR-33b level in stage IV cases is significantly lower than that in stage I, II and III cases. (**D**) GC patients with metastasis have a lower level of miR-33b than those without metastasis (p < 0.01). All data are shown as mean ± SD.

To assess the expression of miR-33b in GC, q-PCR analysis using TaqMan probes was conducted in 150 pairs of clinic GC tissue and matched adjacent normal tissue samples. Among them, 85 cases showed reduced level of miR-33b in tumor tissues compared with their adjacent normal tissues, whereas 65 cases showed up-regulated miR-33b level in GC (Fig. 1A). The results also showed that the average expression of miR-33b in gastric cancer samples was significantly lower than that in the adjacent non-neoplastic tissues (p < 0.05) (Fig. 1B). To further study the relationship between miR-33b expression and clinicopathological factors of GC, the levels of miR-33b in GC tissues (including fully clinical information) were statistically analyzed (non-parametric test). Interestingly, we found that the lower level of miR-33b was associated with pM stage (p = 0.038, metastasis vs non metastasis) and pTNM stage (p = 0.006, stage I vs IV; p = 0.002, stage II vs IV; p = 0.038, stage III vs IV) in GC patients. Moreover, the median level of miR-33b in stage IV cases was significantly lower than that in stage II and III cases (Fig. 1C). GC patients with metastasis also had a lower level of miR-33b than those without metastasis (Fig. 1D). In addition, there was no significant difference between the expression level of miR-33b and other clinicopathologic characteristics, including gender, age, venous invasion, position, borrmann typing, pT stage, pN stage in GC (Supplementary Table S2).

### Overexpression of miR-33b in GC cells inhibits cell proliferation, migration and invasion

**Figure 2:**
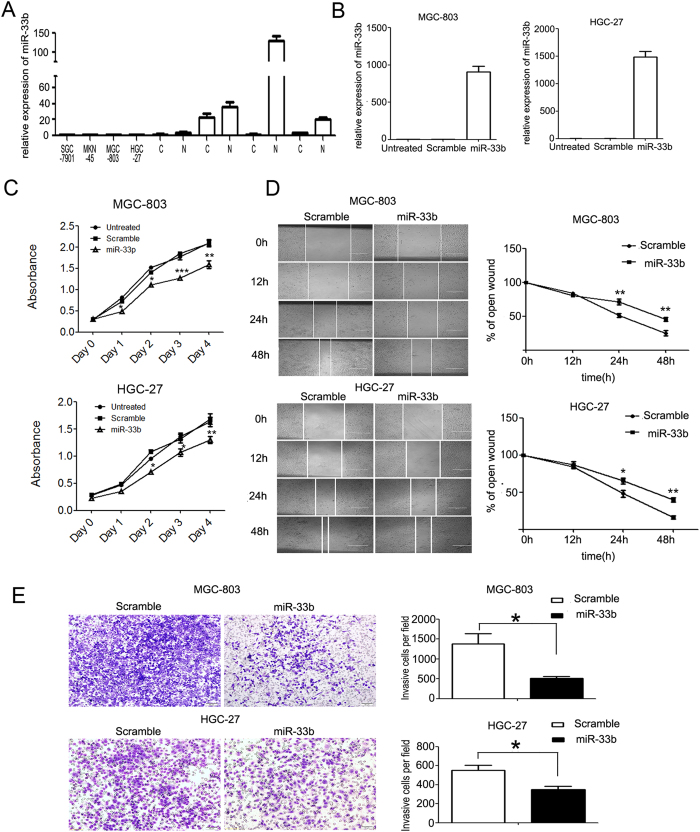
Overexpression of miR-33b in GC cells inhibits cell proliferation, migration and invasion. (**A**) The expression levels of miR-33b in GC cell lines (HGC-27, MGC-803, SGC-7901 and MKN-45) were abnormally down-regulated compared with four paired GC samples. “C” represents clinic GC tissue, “N” represents the matched adjacent normal tissue. (**B**) The expression levels of miR-33b were examined by real-time PCR after transfection with 50 nmol/L of miR-33b mimics or scramble or no transfection. (**C**) The cell growth of MGC-803 and HGC-27 cells at day 0, 1, 2, 3, 4 post transfection which was detected by CCK-8 assay. (**D**) MGC-803 and HGC-27 cells were not transfected or transfected with 50nmol/L of miR-33b mimic or scramble for 24 h, then wounds were made. The relative ratio of wound closure per field was shown. (**E**) MGC-803 and HGC-27 cells were not transfected or transfected with 50 nmol/L of miR-33b mimic or scramble for 24 h, and transwell invasion assay was performed. The relative ratio of invasive cells per field is shown. Magnification for identification of migration and invasion is ×10. Bar, 100 μm. All data are shown as mean ± SD. ^∗^p < 0.05; ^∗^^∗^p < 0.01, ^∗^^∗^^∗^p < 0.001.

To investigate the role of miR-133b in GC carcinogenesis, we firstly measured the level of miR-33b in GC cells and the results showed that miR-33b was significantly down-regulated in MGC-803, HGC-27, SGC-7901 and MKN-45 (Fig. 2A). Of them, the higher malignant cell lines MGC-803 and HGC-27 cells were selected to analyze the role of miR-33b. We re-introduced miR-133b mimics into the two GC cell lines. miR-133b was successfully overexpressed in these two cell lines which was confirmed by q-PCR (Fig. 2B). The overexpression of miR-133b in both of the two GC cell lines can inhibit GC cell proliferation significantly as demonstrated by CCK-8 growth assay. The scramble control had no effect on cell proliferation compared with the untreated cells (Fig. 2C). Cell migration and invasion assays were also performed in MGC-803 and HGC-27 transfected with miR-33b mimics or scramble mimics. Accordingly, the wound healing assay showed that cell migration was inhibited in miR-33b mimic-transfected GC cells compared to the scramble mimic-transfected ones (Fig. 2D), suggesting the inhibitory effects of miR-33b on tumor cell migration. To detect whether miR-33b possesses the ability to inhibit cell invasion, transwell invasion assay was performed. As expected, there was significant reduction in cell invasiveness after miR-33b transfection in both MGC-803 and HGC-27 cell lines (Fig. 2E). These results suggested that miR-33b might act as a tumor suppressor through inhibiting not only cell proliferation but cell metastasis in GC.

### miR-33b targets c-Myc in GC cells and patients

**Figure 3:**
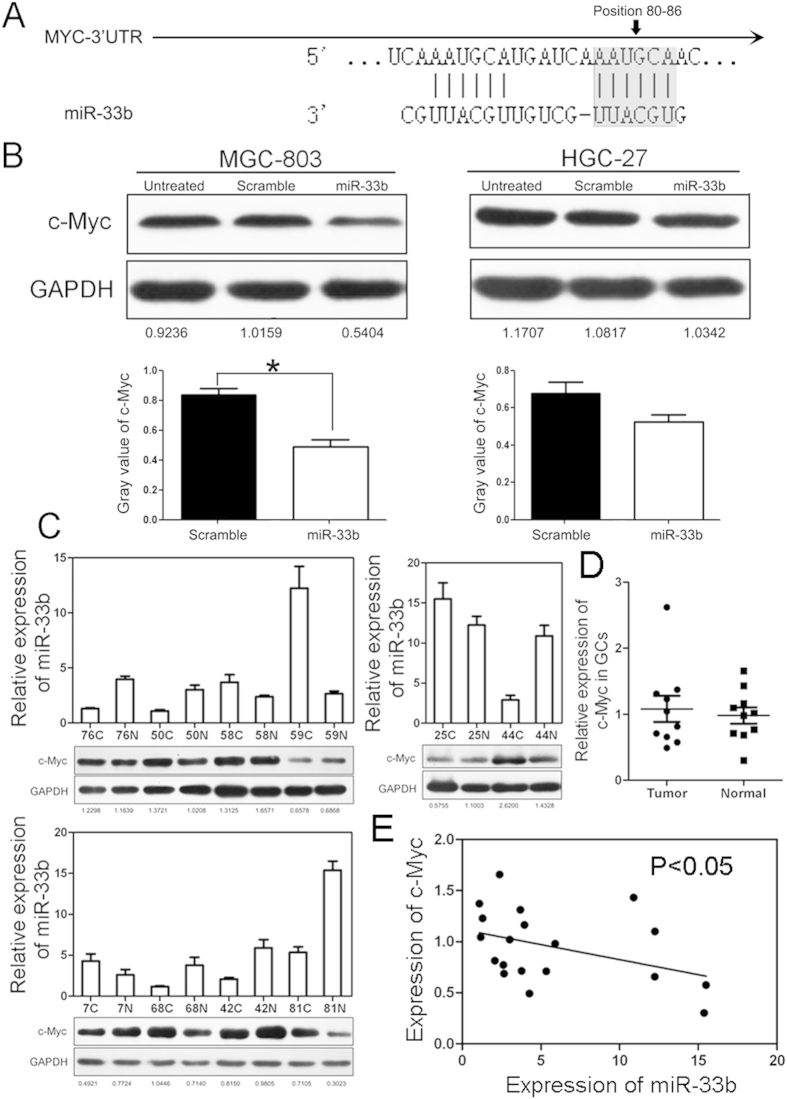
miR-33b targets c-Myc in GC cells and patients. (**A**) Schematic representation of c-Myc 3′ UTRs showing putative miR-33b binding sites. (**B**) Western blot analysis of c-Myc expression in MGC-803 and HGC-27 cells transfected with scramble oligonucleotide or miR-33b mimics (Top). Gray value of c-Myc expression was also shown (Bottom). (**C**) Western blot analysis of c-Myc expression and relative level of miR-33b in 10 pairs of GC tissues. (**D**) Statistical expression of c-Myc in GC samples. (**E**) Expression of miR-33b was negatively correlated with c-Myc in GC sample.

Biological role of miR-33b in GC cells promoted us to study its mechanism in gastric carcinogenesis. To this aim, we started to search for the mRNA targets of miR-33b in gastric cancer. Studies have reported that miR-33b acted as a tumor suppressor by directly targeting oncogene c-Myc^12^. After alignment, we truly found the binding site of miR-33b in the 3′UTR of c-Myc mRNA (Fig. 3A). To validate whether miR-33b targets c-Myc in GC, immunoblotting assay was carried out in GC cells and showed that c-Myc was about 2-fold lower in MGC-803 cells transfected with miR-33b mimics, however there was no obvious change in HGC-27 cells (Fig. 3B). Furthermore, in 10 paired GC samples (only 68C/68N with metastasis) with down-regulated miR-33b, more than half of them showed up-regulated expression of c-Myc. It seemed that the miR-33 level was negatively correlated with expression of c-Myc in GC (Fig. 3C–E). These results indicated that miR-33b might inhibit tumor migration, invasion and proliferation by directly targeting oncogene c-Myc in GC.

### miR-33b is methylation-dependently down-regulated in GC cell lines

**Figure 4:**
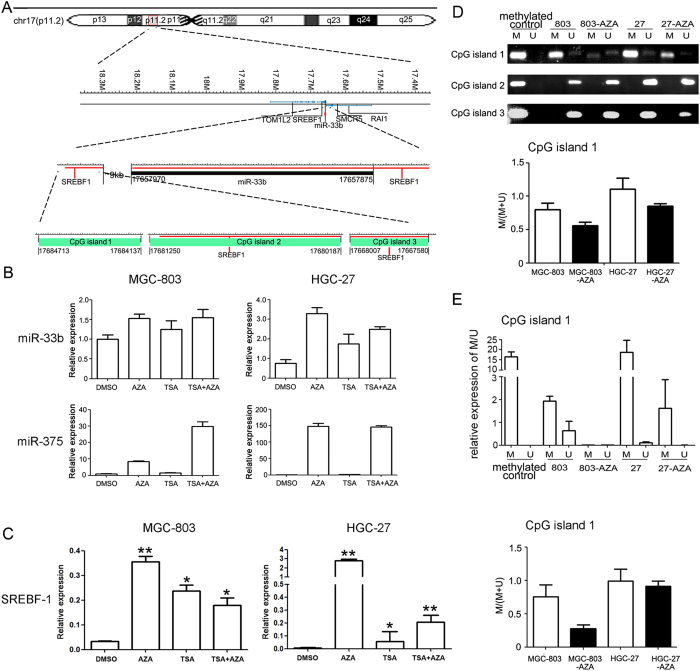
Down-regulation of miR-33b in gastric cancer cells is associated with hypermethylation of miR-33b upstream region. (**A**) Schematic illustration of the CpG islands upstream of miR-33b gene within the chr 17p11.2 segment. (**B**) Effect of 5-Aza-CdR and TSA on miR-33b expression in MGC-803 and HGC-27 gastric cancer cell lines. There is a 1.5-fold increase of miR-33b level after treatment of 5′-AZA (1 μM) also both AZA and TSA, a 1.2-fold increase after treatment of TSA (300 nM) in MGC-803 cells, a 3-fold increase after treatment of 5′-AZA (1 μM), a 2-fold increase after treatment of TSA (300 nM) and 2.5-fold increase after treatment of both AZA and TSA in HGC-27 cells. (**C**) The analysis of SREBF-1 expression in GC cells treated with AZA and/or TSA. There is an obviously increase of SREBF-1 level after treatment of 5′-AZA (1 μM) (p < 0.01) also both AZA and TSA (p < 0.01), a 8-fold increase after treatment of TSA (300 nM) (p < 0.05) in HGC-27 cells, a 12-fold increase after treatment of 5′-AZA (1 μM) (p < 0.01), a 8-fold increase after treatment of TSA (300 nM) (p < 0.05) and 6-fold increase after treatment of both AZA and TSA (p < 0.05) in MGC-803 cells. All data are shown as mean ± SD. ^∗^p < 0.05; ^∗^^∗^p < 0.01. (**D**) The methylation level of the CpG island 1 was decreased in GC cells treated with 5′-AZA, suggesting that CpG island 1 was hypermethylated in MGC-803 and HGC-27 cell lines while CpG island 2 and 3 didn’t have obvious changes in these cells. (**E**) CpG island 1 methylation analysis using qMSP in GC cells. Similar to the results of MSP, the methylation level was decreased in GC cells treated with 5′-AZA. M, methylated state; U, unmethylated state; Methylated control, Methyltransferase treated normal lymphocytes DNA.

Given that miR-33b is an important regulator in GC, we planned to analyze the regulatory mechanism of miR-33b expression. Since hypermethylation is a general event in carcinogenesis, we initially analyzed the genome locus of miR-33b gene and identified three CpG islands upstream of it (Fig. 4A). To find out the cause of the lower expression of miR-33b in GC, we first examined the expression of miR-33b in 5′-AZA and/or TSA treated GC cells. The results showed that there was a 1.5-fold increase of miR-33b level after treatment of 5′-AZA also both AZA and TSA, a 1.2-fold increase after treatment of TSA in MGC-803 cells, a 3-fold increase after treatment of 5′-AZA, a 2-fold increase after treatment of TSA and a 2.5-fold increase after treatment of both AZA and TSA in HGC-27 cells (Fig. 4B), suggesting that miR-33b expression in GC cells might be regulated by DNA methylation. Since miR-375 has been demonstrated to be epigenetically inactivated in GC, we used it as a positive control here^13^. Meanwhile, to investigate whether miR-33b’s host gene-SREBF1 was regulated by DNA methylation, we also examined the level of SREBF1 and found that there was significant up-regulated SREBF1 expression in 5′-AZA treated MGC-803 and HGC-27 cells, suggesting that both miR-33b and its host gene could be silenced together by the hypermethylation of CpG island (Fig. 4C).

Furthermore, we detected the DNA methylation status using MSP and q-MSP in MGC-803 and HGC-27 cells, the assay revealed that CpG island 1 upstream of miR-33b had higher methylation level in MGC-803 and HGC-27 cells, which was consistent with the lower expression of miR-33b in these GC cell lines. On the contrary, CpG island 2 and 3 didn’t have obvious changes in these cells (Fig. 4D,E).

### Methylation analysis of miR-33b CpG islands in GC cases

**Figure 5:**
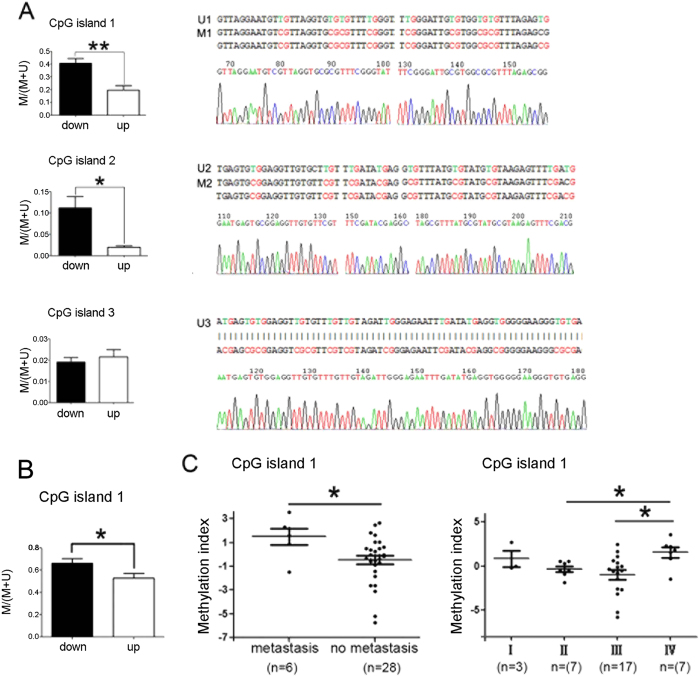
The methylation analysis of miR-33b regulatory regions in GC cases. (**A**) The methylation status in CpG island 1/2/3. The methylation degree of CpG island 1 in down-regulation group was obviously higher than that in up-regulation group (p < 0.01); The methylation degree of CpG island 2 in down-regulation group was obviously higher than that in up-regulation group (p < 0.05). The unmethylated state of CpG island 3 is higher than methylated state in this island. M, methylated state; U, unmethylated state. All data are shown as mean ± SD. ^∗^p < 0.05; ^∗^^∗^p < 0.01. (**B**) The methylation status of CpG island 1 analyzed by using qMSP. Similar to the results of MSP, the methylation degree in down-regulation group was higher than that in up-regulation group (p < 0.05). (**C**) Correlation analysis of methylation index with pM stage or pTNM in GC tissues. The methylation index in stage IV cases is higher than that in stage II and III cases (p < 0.05). The methylation index in GC patients with metastasis is also higher than those without metastasis (p < 0.05). All data are shown as mean ± SD. ^∗^p < 0.05.

To determine whether aberrant hypermethylation is responsible for down-regulation of miR-33b expression in GC patients, we next examined the methylation status in CpG island 1/2/3 upstream of miR-33b. MSP assay was performed in 42 paired GC samples, including 21 patients with lower miR-33b levels (down-regulation group) and 21 patients with higher miR-33b levels (up-regulation group). We analyzed the methylation status of three CpG islands upstream of miR-33b in a group of primary GC samples and adjacent normal tissue, respectively. Our data showed DNA methylation of CpG island 1/2 upstream of miR-33b existed in both adjacent normal tissues and cancer tissues, but, the overall methylation degree in down-regulation group was obviously higher than that in up-regulation group (p < 0.05) (Fig. 5A). However, there was no difference in overall methylation degree of CpG island 3 between the down-regulation group and up-regulation one (Fig. 5A). Meanwhile, we observed that DNA methylation was more frequent in CpG island 1/2 than CpG island 3, which was partially different with that in GC cell lines (Supplementary Figure S1A-C). Of them, one sample was randomly selected for sequencing, respectively (Supplementary Figure S2).

### Methylation index analysis of miR-33b CpG island 1 in GC cases

To further analyze the methylation status of the CpG island 1, q-MSP was subsequently performed and specific primers were first tested. Similar to the results of MSP analysis, the methylation degree in down-regulation group was significantly higher than that in up-regulation group (p < 0.05) (Fig. 5B; Supplementary Table S3). Next, the methylation index of GC cases was calculated and its correlation with pM stage and pTNM of GC tissues was analyzed. The methylation index in stage IV cases is significantly higher than that in stage II and III cases (p < 0.05), respectively. Moreover, methylation index in GC patients with metastasis is also higher than those without metastasis (p < 0.05) (Fig. 5C). The data suggested that the hypermethylation of the CpG islands upstream of miR-33b might lead to the low expression of miR-33b in GC and further possibly resulted in aberrant cell migration and invasion during gastric carcinogenesis.

### miR-33b inhibits tumorigenicity in nude mice

**Figure 6:**
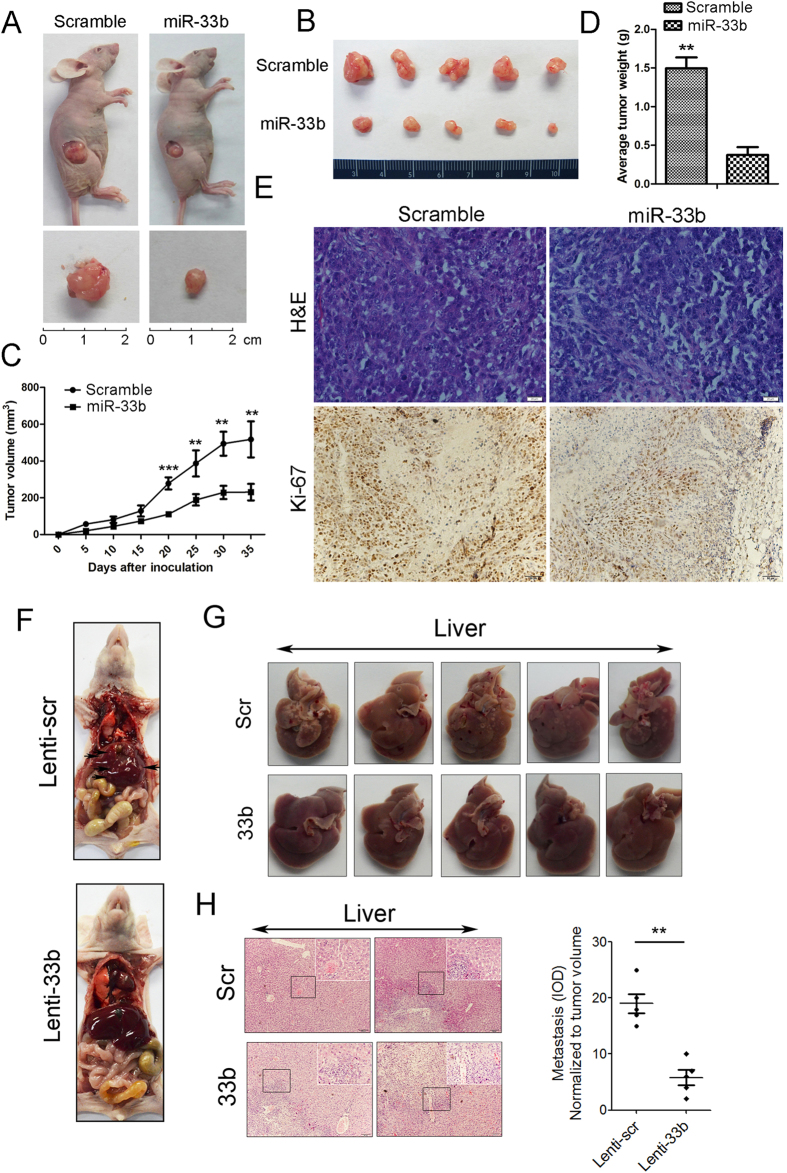
miR-33b suppresses gastric tumorigenicity *in vivo*. (**A**) Photographs of the mice injected with scramble control and miR-33b mimics. (**B**) Tumors formed in the two group of nude mice. 5 × 10^6^ HGC-27 cells were subcutaneously injected into nude mice and miR-33b mimics (or scramble control) were directly into the tumor every 7 days. Tumors were harvested after 5 weeks. (**C**) Graph representing tumor volumes at the indicated days during the experiment for the miR-33b mimics group and scramble group. Five mice in each group. (**D**) Tumor weight averages between the scramble and miR-33b mimics groups at the end of the experiment (day 35). (**E**) H&E staining and immunohistochemistry analysis of Ki-67 expression in tumors from xenograft mice of the two group. (**F**) Nude mice were injected with HGC-27 cells infected with Lenti-33b or Lenti-scr through the lateral tail vein. Five weeks after injection, the mice were killed and the livers were dissected for microscopic histology. (**G**) The numbers of liver metastases in mice injected with Lenti-33b-infected HGC-27 cells were significantly lower than those in mice injected with Lenti-scr-infected cells. (**H**) Histological analysis of sections from livers of the mice injected with HGC27 cells treated by either Lenti-scr (control) or Lenti-33b. Images shown in the top-right panel represent magnified view of boxed region indicated in the panel. Data are mean ± SD and representative of three independent experiments. (^∗^p < 0.05; ^∗^^∗^p < 0.01; ^∗^^∗^^∗^p < 0.001).

Finally, we investigated whether miR-33b could suppress gastric tumorigenicity *in vivo*. About 5 × 10^6^ HGC-27 cells were injected subcutaneously in posterior flanks of immunocompromised nude mice and then miR-33b mimics (or scramble control) were directly injected into the tumors. After 5 weeks, the tumors were harvested and it was noteworthy that tumors formed in the miR-33b group were much smaller than those from the scramble group (Fig. 6A,B). In agreement with the tumor growth curve, the volumes and weights of tumors treated by miR-33b mimics were significantly lower than scramble control-injected tumors. In view of these observations, we speculated that ectopic expression of miR-33b might suppress tumor cell proliferation. To address this point, immunohistochemical analysis was performed to measure the protein levels of Ki-67 in the tumor tissues. The data showed that miR-33b mimics injection decreased expression of Ki-67 in the tumor tissues (Fig. 6E). We further conducted metastasis assay *in vivo*. In doing so, 1 × 10^6^ viable HGC-27 cells infected with Lenti-33b or Lenti-scr were re-suspended in 0.1 ml phosphate-buffered saline and injected into the lateral tail vein. Five weeks after injection, we sacrificed the mice and dissected the livers for microscopic histology. The numbers of liver metastasis in mice that were injected with Lenti-33b-infected HGC-27 cells were significantly lower than those in mice injected with Lenti-scr-infected cells (Fig. 6F–H). Immunohistochemistry was performed to assess the pathological properties of the liver tissues and showed more metastatic nodules in Lenti-scr treated mice than Lenti-33b treated ones (Fig. 6H). These data indicated that re-introduction of miR-33b obviously inhibited the metastasis of GC cells *in vivo*. Taken together, these data indicated that re-introduction of miR-33b significantly inhibited the gastric tumorigenicity in the nude mouse xenograft model.

## Discussion

Emerging evidence indicates that miRNAs often act as oncogenes or tumor suppressors to regulate many cellular events in different steps of tumor formation and progression^5^,^14^. Some small molecules are found to be able to modulate miRNA biogenesis and miRNA-mediated gene regulation, even several molecules of these can inhibit tumor growth through controlling miRNA processing and maturation, representing the huge potential of targeting miRNAs in cancer therapy^15^,^16^,^17^,^18^. Although a few miRNAs have been well studied, the role and molecular mechanism of many other miRNAs were not well known in gastric cancer. In this work, the expression of miR-33b was analyzed in 150 GC cases from several hospitals and showed down-regulated tendency in more than 55% patients. The expression level of miR-33b in GC tissues was also significantly lower than that in adjacent non-neoplastic tissues. Moreover, when we analyzed the correlation between miR-33b levels and clinicopathological information of GC patients, we found that miR-33b gradually decreased in stage II/III/IV of GC patients. Furthermore, we found that miR-33b level was significantly down-regulated in patients with metastasis than that without metastasis. These data suggested that miR-33b might be not necessarily linked with the occurrence of GC, while it is possibly correlated with tumor progression and metastasis.

Our clinicopathological analysis promoted us to analyze the biological function of miR-33b in GC cells. 4 GC cell lines were initially assessed. Of them, HGC-27 is an undifferentiated GC cell line, MGC-803 and MKN-45 are poorly differentiated GC cell lines, and SGC-7901 is a moderately differentiated GC cell line. Since the miR-33b level in 4 GC cell lines is significantly lower than that in normal gastric tissues, we chose the higher malignant cell lines to study the roles of miR-33b. As expected, the data in miR-33b mimic treated MGC-803 and HGC-27 cells showed strongly inhibitory roles of miR-33b in cell migration, invasion and growth, demonstrating that miR-33b acted as a tumor suppressive gene *in vitro*. More importantly, re-introduction of miR-33b could obviously inhibit gastric tumorigenesis and suppress tumor growth *in vivo*, which strengthened our conclusion that miR-33b function as a tumor suppressor in GC.

We next investigated the mechanism that miR-33b acted as a tumor suppressor in GC. Oncogene c-Myc is an important member of the MYC gene family and is also a transcription factor that has been implicated in the regulation of up to one-third of human genes^19^,^20^, placing it at the center of many biological processes, including cell cycle, differentiation and proliferation^21^. One previous report indicated that miR-33b directly inhibited c-Myc expression in medulloblastoma cells, suggesting that miR-33b might act as a tumor suppressor through targeting c-Myc in gastric cancer^12^. In this work, MGC-803 and HGC-27 cells were transfected with miR-33b mimics and immunoblotting was performed to analyze the expression of c-Myc. We are cognizant that miR-33b negatively regulates the expression of c-Myc in GC cells. However, an analysis of c-Myc protein levels in 10 cancer tissues with down-regulated miR-33b indicated that only ~50% samples showed up-regulated c-Myc. We deduced that there might be other regulatory factors to modulate the protein level of c-Myc in gastric carcinogenesis and more samples need to be further studied.

Meanwhile, we investigated the possibly regulatory mechanism of miR-33b expression in gastric cancer. Methylation and acetylation were first thought because they have been proved to play critical roles in many cancers^22^,^23^,^24^,^25^. Through the expression analysis of miR-33b in MGC-803 and HGC-27 cells treated with AZA and/or TSA, we highly suspected methylation was one of the regulatory mechanisms of miR-33b expression. DNA hypermethylation of CpG sites within CpG islands was known as an epigenetic aberration leading to the inactivation of tumor-suppressive genes in cancer cells^26^. Recent studies clearly demonstrated DNA methylation-mediated down-regulation of tumor-suppressive miRNA gene expression in various types of cancers^27^,^28^,^29^. Here, we found three CpG islands upstream of miR-33b gene. Of them, CpG island 1 was in the extragenic region, CpG island 2 and 3 were located within the SREBF1 gene. Aberrant expression of miR-33b in GC maybe result from long-range control of part of 3 CpG islands or collaboration of them. It was necessary to validate the proposed mechanisms above. Therefore, MSP and qMSP analysis were performed to analyze the accurate status of DNA methylation in CpG islands 1/2/3, and indicated that the hypermethylation of CpG island 1/2 might regulate the expression of miR-33b in gastric cancer. Correspondingly, CpG island 3 probably did not work or just play synergetic roles.

In summary, our results indicated that deregulation of miR-33b in GC was associated with pM stage and pTNM stage and the expression of miR-33b could be negatively regulated by aberrant DNA methylation. Furthermore, reintroduction of miR-33b suppressed gastric tumorigenicity via targeting c-Myc *in vitro* and *in vivo*. All of these data suggest that miR-33b might act as a tumor suppressor in GC.

## Materials and Methods

### Patients and GC cell lines

Paired samples were obtained from patients undergoing surgery for gastric cancer from The General Hospital of the People’s Liberation Army and Cancer Institute and Hospital, Chinese Academy of Medical Sciences. The samples were immediately frozen and stored in liquid nitrogen until RNA extraction. The use of the tissue samples for all experiments was approved by the ethical board of the Institute of Basic Medical Sciences, Chinese Academy of Medical Sciences (IBMS, CAMS). Specifically, all participants provided their verbal informed consent to participate in this study and were compensated for some money. Their verbal informed consents were written down. This consent process was discussed and approved by the ethics board. We collected the clinical samples in accordance with the approved guidelines.

The MGC-803, HGC-27, MKN-45, and SGC-7901 cell lines were purchased from the Cell Resource Center of IBMS, CAMS. MGC-803 was propagated in DMEM (Life Technologies, CA, USA), supplemented with 10% fetal bovine serum (FBS; PAA, Pasching, Austria) and streptomycin (100 μg/ml), penicillin (100 U/ml). The HGC-27, MKN-45, SGC-7901 were maintained in RPMI 1640 medium (PAA) supplemented with 10% FBS (PAA).

### RNA extraction and quantitative real-time PCR (q-PCR)

Total RNA was extracted from the cells and tissues using Trizol reagent (Invitrogen, CA, USA), according to the manufacturer’s instructions. Real-time PCR assay was conducted to detect the level of miR-33b. Briefly, cDNA was synthesised by M-MLV reverse transcriptase (Invitrogen) from 5 ug of total RNA. Stem-poop RT primer was used for the reverse transcription of miR-33b.

Quantitative RT-PCR was performed on the Bio-rad CFX96 real-time PCR System (Bio-rad, Foster City, CA, USA) using KAPA PROBE FAST qPCR Kits (Kapa Biosystems, MA, USA) and TaqMan probes (Invitrogen) with the following cycling conditions: 95 °C for 10 min (initial denature); then 40 cycles of 95 °C for 15 sec, 60 °C for 60 sec. The miR-33b specific forward primer sequence was designed on the basis of miRNA sequences obtained from the miRBase database. Human U6 snRNA was used for normalization. Related primer sequences were provided in Supplementary Table 1.

### Transfections, reagents and assays

The miR-33b mimics with sequence of 5′-GUGCAUUGCUGUUGCAUUGC-3′, and negative control RNA-scramble oligonucleotides with sequence of 5′-UUCUCCGAACGUGUCACGUTT-3′, were gained from RIBOBIO (Guangzhou, China). The day before transfection, GC cells were seeded onto 24-well plates (1 × 10^5^ cells per well) in serum-free medium. Transfection was carried out using Lipofectamine 2000 (Invitrogen) with miR-33b mimics (50 nmol/L) or scramble (50 nmol/L) accordance with the manufacturer’s procedure (Invitrogen). The expression level of miR-33b mimics in GC cells was assayed by real-time PCR. For Western blots and other functional analysis, Cells (≈70% confluent) were transfected with miR-33b mimics or scramble using Lipofectamine 2000. HGC-27 and MGC-803 cells were grown in normal culture medium containing 50 nmol/L miR-33b or scramble control for indicated time points.

### Cell proliferation, migration and invasion assays

For cell proliferation assay, cells were incubated in 10% CCK-8 (Dojindo, Kumamoto, Japan) diluted in normal culture medium at 37 °C until visual color conversion occurred. Proliferation rates were determined at each time points after transfection. For cell migration assay, GC cells were seeded onto 24-well plates (1 × 10^5^ cells per well) 24 hours after transfection, then cells are maintained in medium containing 10% fetal bovine serum at 37 °C. After cell confluence, an artificial wound was created using a 200-μL pipette tip, then cells were washed twice with PBS and were cultured in medium containing 1% fetal bovine serum at 37 °C. To visualize migrated cells and wound healing, images were taken at 0, 12, 24, 36, 48, and 60 hours. Matrigel-coated membrane matrix (MILLIPORE, Darmstadt, Germany) is used to evaluate the ability of cell passing. GC cells were seeded onto a Matrigel-coated membrane matrix prior paved with ECM (BD Biosciences, San Jose, CA, USA) 24 hours after transfection. Fetal bovine serum was added to the lower chamber as a chemoattractant. After 24 hours, the non-invading cells were removed. Invasive cells located on the lower surface of the chamber were stained with May-Grünwald and Giemsa and counted.

### Western blotting

Western blot analysis was carried out using standard methods. Proteins were separated by 10% SDS-PAGE, and transferred onto PVDF membranes (Millipore Corporation, Billerica MA, USA). Membranes were blocked overnight with 5% non-fat dried milk for 2 h and incubated with anti-c-Myc antibody (Bioworld) at 1:2000 dilution; anti-GAPDH antibody (Proteintech) at 1:50,000 dilution overnight at 4 °C. After washing with TBST (10 mM Tris, pH 8.0, 150 mM NaCl, and 0.1% Tween20), the membranes were incubated for 2 h at room temperature with goat anti-rabbit antibody (Zsgb-bio, Beijing, China) at 1:20000.

### Drug treatment

GC cell lines were seeded at a concentration of 1 × 10^5^ cells per well 24 hours prior to treatment with 5-aza-2′deoxycytidine (AZA) (Sigma) and/or trichostatin A (TSA) (Sigma). For AZA treatment, cells were continuously administered by replacing the medium containing 1 μM AZA every 24 hour for 3 days. For TSA treatment, 300 nM TSA was added after 48 hours. For both AZA and TSA treatment, AZA was removed after 48 hours, then TSA was added, and cells were collected after 72 hours. Medium containing DMSO was used as a positive control.

### Methylation-specific PCR (MSP)

Sequence analysis upstream of miR-33b gene was performed in the human genome database and three CpG Islands around miR-33b are found. Methyl rimer Express v1.0 was used to design methylation specific PCR (MSP) primers, which were provided in Supplementary Table 1. Genomic DNA samples were amplificated by PCR after modified by sodium bisulfite using the EpiTect Bisulfite Kit (QIAGEN) following the manufacturer’s instructions. Normal lymphocyte DNA treated by Methyltransferase (NEB) was used as a positive control and the reaction system without any template was used as blank/negative control. PCR products were subjected to gel electrophoresis through a 3% agarose gel and were visualized by ethidium bromide staining and UV transillumination. The DNA methylation status was analyzed by ImageJ.

### Quantitative methylation-specific PCR (qMSP) and methylation index

The sodium bisulfite-treated DNA was amplificated on the Bio-rad CFX96real-time PCR System (Bio-rad) using KAPA SYBR FAST qPCR Kits (Kapa Biosystems) with the following cycling conditions: 95 °C for 10 min (initial denature); then 40 cycles of 95 °C for 15 sec, 60 °C for 60 sec. To correct the differences in both quality and quantity between samples, GAPDH was used as an internal control. QMSP was performed 3 times on each sample and related primer sequences were provided in Supplementary Table 1. Methylation index = logC/N, 1.5; C = MC/(MC + UC), N = MN/(MN + UN), where M is the copy number of methylated CpG island 1, U is the copy number of unmethylated CpG island 1.

### Analysis of the effect of miR-33b in nude mice

Animal xenograft model studies were carried out in accordance with the approved guidelines; 5 × 10^6^ HGC-27 cells were injected subcutaneously into the posterior flanks of 6-week-old female nude mice, five mice per group. Then, miR-33b mimics and scrambled control diluted in Lipofectamine 2000 (Invitrogen) solution (100 nmol mimics in 100 μl total volume) were injected directly into the tumors, respectively. The tumors were injected every 7 days for a total of 5 times. Tumor diameters were measured every 5 days. At 35 days after injection, mice were killed and tumors were weighted after necropsy. Tumor volume was monitored by measuring the length (L) and width (W) of the tumor with calipers and was calculated with the formula V = (L×W^2^) ×0.5.

Meanwhile, we estimate the effect of miR-33b in regulating metastatic ability of gastric cancer cells *in vivo*. Lenti-33b- and Lenti-scr-infected HGC27 cells (1 × 10^6^) were suspended in 0.1 ml phosphate-buffered saline and injected into the lateral tail vein. Five weeks after injection, mice were killed and the livers were extracted and fixed in 4% paraformaldehyde in phosphate-buffered saline. Paraffin embedding, sectioning and staining with hematoxylin–eosin were performed. Visible liver metastases were measured and counted using a microscope.

### Histology and immunohistochemistry

Mouse tumor tissues were fixed with 10% formalin for 24 h, embedded in paraffin and cut into 3 μm-thick sections. These paraffin sections were stained with hematoxylin (H) for 90 s and eosin (E) for  min to observe cell morphology. Immunohistochemical staining was carried out on the paraffin-embedded sections. In short, slides were retrieved with citrate buffer in a steam pressure cooker for 90 s. Endogenous peroxidase activity was quenched with 3% hydrogen peroxide and the slides were incubated in goat serum for 30 min at room temperature. Ki-67 (Dako, Denmark) were applied at 1:100 dilution in PBS at 4 °C overnight. The slides were washed with PBS and incubated with horseradish peroxidase–conjugated anti-rabbit kit (Dako, Denmark). All slides were counterstained with hematoxylin photographed with optical microscopy Olympus BX51.

All of the above experimental protocols were approved by the committee of Institute of Basic Medical Sciences, Chinese Academy of Medical Sciences (IBMS, CAMS).

## Additional Information

**How to cite this article**: Yin, H. *et al.* DNA Methylation mediated down-regulating of MicroRNA-33b and its role in gastric cancer. *Sci. Rep.* **6**, 18824; doi: 10.1038/srep18824 (2016).

## Supplementary Material

Supplementary Information
