# Supplementary material for: DNA Methylation mediated down-regulating of MicroRNA-33b and its role in gastric cancer
Source: Sci Rep. 2016 Jan 5;6:18824. doi: 10.1038/srep18824 (PMC4700416; doi:10.1038/srep18824)
Supplement: Supplementary Information [file srep18824-s1.pdf]

DNA Methylation mediated down-regulating of MicroRNA-33b and its role in gastric cancer

Haixin Yin[1, 2, 6], Peng Song[3, 6], Rui Su[1, 6],Guihua Yang[4, 6], Lei Dong[1], Min Luo[1], Bin Wang[1, 2], Bei Gong[4], Changzheng Liu[1], Wei Song[1], Fang Wang[1], Yanni Ma[1], Junwu Zhang[1], Weibin Wang[5 *], Jia Yu[1*]

[1]Department of Biochemistry, Institute of Basic Medical Sciences, Chinese Academy of Medical Sciences (CAMS) & Peking Union Medical College (PUMC), Beijing 100005, PR China

[2]Department of Surgery，The Shanxi Academy of Medical Sciences & Shanxi Dayi Hospital, Taiyuan 030032, PR China

[3]The First Surgery Department of Nanlou, Chinese People's Liberation Army General Hospital, Beijing 100853, PR China

[4]Department of Clinical Laboratory, Beijing Shijitan Hospital, Beijing100038, PR China

[5]Department of General Surgery, Peking Union Medical College Hospital, CAMS & PUMC, Beijing 100005, PR China

[6]These authors contributed equally to this work.

[*] To whom correspondence should be addressed. E-mail: j-yu@ibms.pumc.edu.cn, Fax: 86-10-65240529, Tel: 86-10-69156423; wwb_xh@yahoo.com.cn; Tel: 86-10-69156007; Fax: 86-10-69156007

Supplementary Figures:

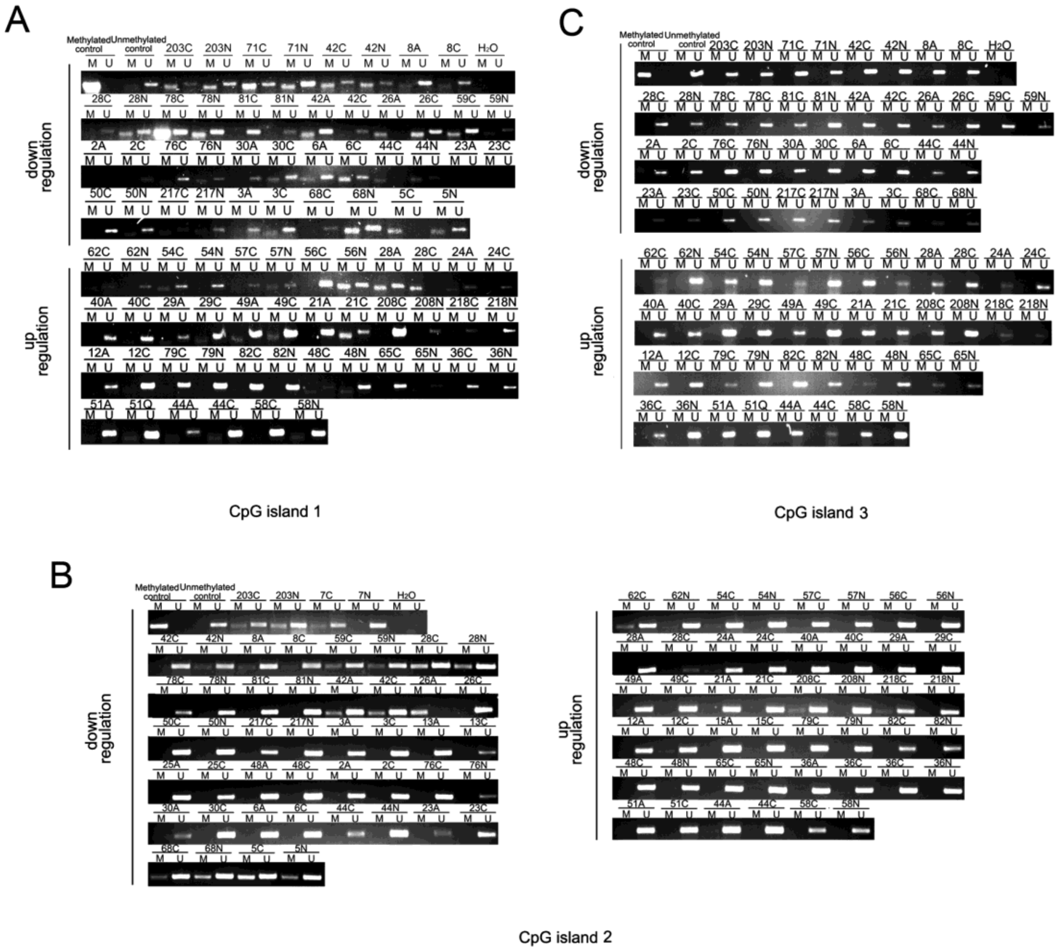

Supplementary Figure 1**.** Agarose gel electrophoresis of MSP analysis of miR-33b CpG islands in GC tissues. M, methylated state; U, unmethylated state; Methylated control, Methyltransferase treated normal lymphocytes DNA; Unmethyl control, normal lymphocytes DNA.

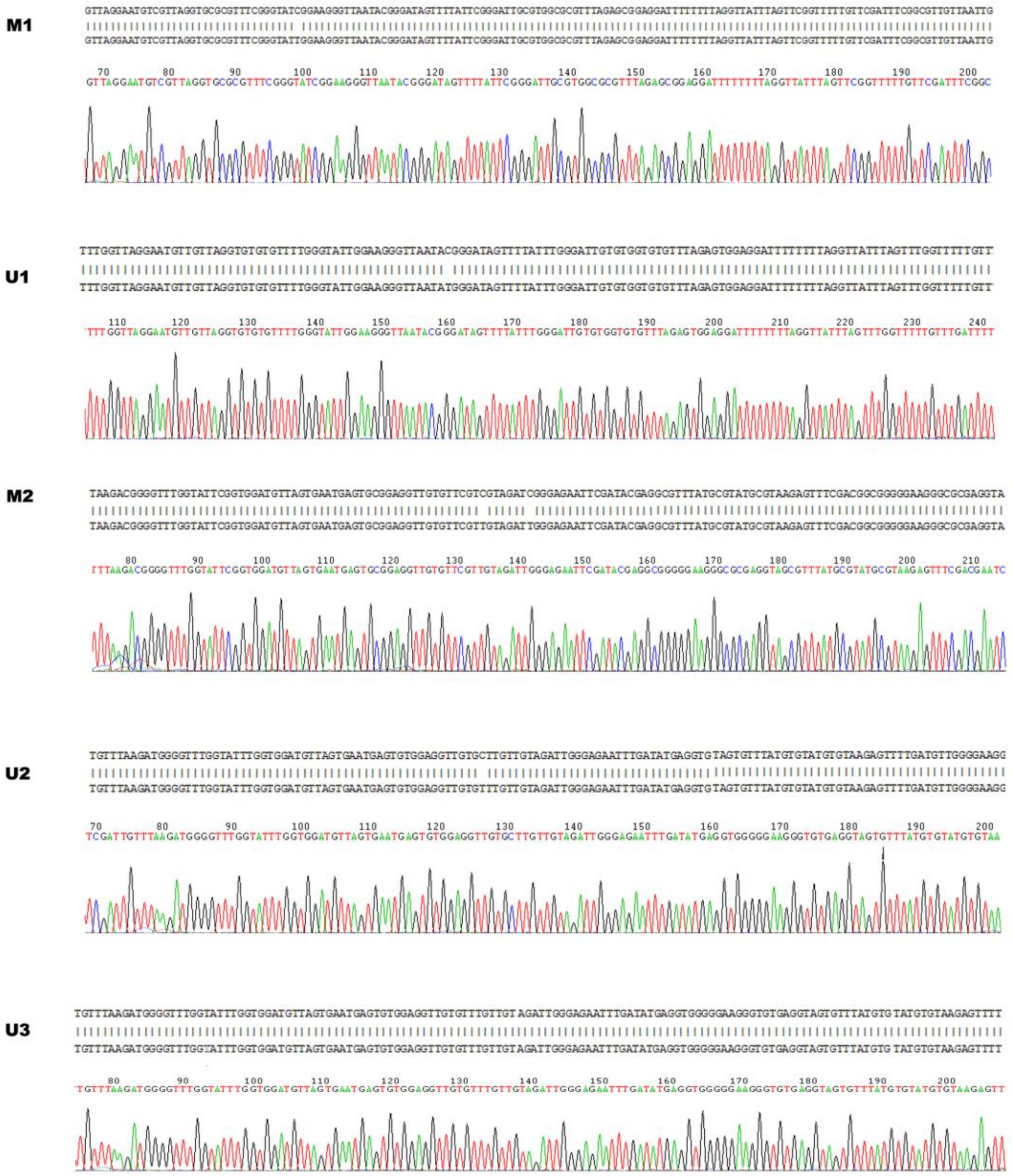

Supplementary Figure 2. The sequencing data of CpG island 1/2/3 in one GC sample.

Table S1. The primers used in the article.

	primer	sequence (5'-3')
**Real-time PCR**	miR-33b RT	GTCGTATCCAGTGCAGGGTCCGAGGT<br>ATTCGCACTGGATACGACTGCAATGC
	miR-33b forward	GCGTGCATTGCTGTTGCAT
	miR-33b reverse	GTGCAGGGTCCGAGGT
	miR-33bTaqMan probe	FAM-CTGGATACGACTGCAATGC-MGB
	U6 RT	AAAATATGGAACGCTTCACGAATTTG
	U6 forward	CTCGCTTCGGCAGCACATATACT
	U6 reverse	ACGCTTCACGAATTTGCGTGTC
	U6 probe	FAM-CCATGCTAATCTTCTCTGTA-MGB
	SREBF-1 forward	TTGCCGACCCTGGTGAGT
	SREBF-1 reverse	AATGGCGTTGTGGGCTGT
**MSP**	M1 forward:	GTTAGGAATGTCGTTAGGTGC
	M1 reverse:	CAATTAACAACGCCGAAAT
	U1 forward:	TTGGTTAGGAATGTTGTTAGGTGT
	U1 reverse:	TCCCAATTAACAACACCAAAAT
	M2 forward:	TAAGACGGGGTTTGGTATTC
	M2 reverse:	CGTCGAAACTCTTACGCATA
	U2 forward:	GTTTAAGATGGGGTTTGGTATTT
	U2 reverse:	AACATCAAAACTCTTACACATAC
	M3 forward:	TTGTCGTTTCGTTAGGGTTC
	M3 reverse:	TTAAAAACGAACGTCCGCTA
	U3 forward:	GTTGTTGTTTTGTTAGGGTTT
	U3 reverse:	ATTAAAAACAAACATCCACTA
**QMSP**	M1 forward:	GTTAGGAATGTCGTTAGGTGC
	M1 reverse:	CAATTAACAACGCCGAAAT
	U1 forward:	TTGGTTAGGAATGTTGTTAGGTGT
	U1 reverse:	TCCCAATTAACAACACCAAAAT
	GAPDH forward:	CGCTTTCTTTCCTTTCGC
	GAPDH reverse:	TGCCCATTCATTTCCTTCC

Table S2. Assiociation analysis between the expression of miR-33b with clinicopathological features in patients with GC.

	N	mean	SD	pValue
**Gender**				
Male	121	-0.692	4.675	
Female	29	1.020	3.278	0.190
**Age**				
>= 60	77	-0.568	3.812	
< 60	73	0.332	5.328	0.227
**Venous invasion**				
Negative	70	-0.389	4.156	
Positive	54	-0.223	4.145	0.936
**Position**				
Cardia	41	-0.409	4.378	
Gastric body	45	-0.021	4.823	
Gastric antrum	39	-0.612	4.411	0.904
**Borrmann typing**				
Ⅰ	98	-0.354	4.323	
Ⅱ	8	-1.433	4.418	
Ⅲ+Ⅳ	7	-0.213	4.456	0.834
**pT stage**				
T1+T2	24	-0.122	5.919	
T3+T4	113	-0.362	4.187	0.845
**pN stage**				
N0+N1	95	0.038	4.023	
N2+N3	33	-0.154	5.321	0.877
**pM stage**				
M0	123	0.018	4.423	
M1	14	-3.189	4.763	0.035*
**pTNM stage**				
Ⅰ	27	1.235	4.351	
Ⅱ	35	1.014	3.340	
Ⅲ	64	0.035	3.157	
Ⅳ	24	-3.351	3.223	0.008**

* p<0.05 **p<0.01

	A	B	C	D	E	F	G	H	I	J	K
1		**Table S3. Raw data of qMSP analysis in GC tissue**									
2		Data Set	Target	Sample	Relative Quantity	Relative Quantity SD	Corrected Relative Quantity SD	Relative Quantity SEM	Correcte d Relative Quantity	Unscaled Expression	Unscaled Expression SD
3		1-SYBR	methylation	Methylation control	0.21601	0.02921	0.02921	0.02065	0.02065	16.63273	2.24876
4		1-SYBR	unmethylation	Methylation control	0.00190	0.00044	0.00044	0.00025	0.00025	0.00066	0.00095
5		1-SYBR	methylation	803	1.00000	0.01649	0.01649	0.01166	0.01166	1.92858	0.22276
6		1-SYBR	unmethylation	803	1.00000	0.31052	0.31052	0.17928	0.17928	1.00000	0.33387
7		1-SYBR	methylation	803 AZA	0.00194	0.00106	0.00106	0.00061	0.00061	0.00263	0.00211
8		1-SYBR	unmethylation	803 AZA	0.00190	0.00044	0.00044	0.00025	0.00025	0.00366	0.00095
9		1-SYBR	methylation	27	0.31118	0.09645	0.09645	0.06820	0.06820	18.76637	5.81640
10		1-SYBR	unmethylation	27	0.00033	0.00000	0.00000	0.00000	0.00000	0.32044	0.00000
11		1-SYBR	methylation	27 AZA	0.50127	0.05458	0.05458	0.03151	0.03151	1.10673	0.87671
12		1-SYBR	unmethylation	27 AZA	0.00194	0.00106	0.00106	0.00061	0.00061	0.00263	0.00211
13		1-SYBR	methylation	203C	0.01515	0.02473	0.02473	0.01428	0.01428	0.12595	0.20564
14		1-SYBR	methylation	203N	0.00033	0.00000	0.00000	0.00000	0.00000	0.32044	0.00000
15		1-SYBR	methylation	42C	0.05854	0.03096	0.03096	0.01787	0.01787	0.05854	0.03178
16		1-SYBR	methylation	42N	0.12166	0.09184	0.09184	0.05303	0.05303	0.26861	0.29247
17		1-SYBR	methylation	71C	0.21601	0.02921	0.02921	0.02065	0.02065	16.63273	2.24876
18		1-SYBR	methylation	71N	0.10121	0.09303	0.09303	0.05371	0.05371	0.15125	0.16713
19		1-SYBR	unmethylation	203C	0.27136	0.06854	0.06854	0.03957	0.03957	2.25608	0.56982
20		1-SYBR	unmethylation	203N	0.00027	0.00040	0.00040	0.00023	0.00023	0.26372	0.39471
21		1-SYBR	unmethylation	42C	1.00000	0.31052	0.31052	0.17928	0.17928	1.00000	0.33387
22		1-SYBR	unmethylation	42N	0.50127	0.05458	0.05458	0.03151	0.03151	1.10673	0.87671
23		1-SYBR	unmethylation	71C	0.13184	0.00982	0.00982	0.00567	0.00567	10.15168	0.75643
24		1-SYBR	unmethylation	71N	0.58206	0.18858	0.18858	0.10888	0.10888	0.86984	0.60324
25		1-SYBR	methylation	208C	0.64768	0.03408	0.03408	0.02410	0.02410	7.50335	0.39486
26		1-SYBR	methylation	208N	0.31847	0.03444	0.03444	0.02435	0.02435	6.95616	1.67017
27		1-SYBR	methylation	218C	0.69737	0.06995	0.06995	0.04038	0.04038	9.98282	1.50565
28		1-SYBR	methylation	218N	0.70281	0.27787	0.27787	0.19648	0.19648	5.11976	2.02421
29		1-SYBR	methylation	21A	0.04047	0.01234	0.01234	0.00873	0.00873	0.57670	0.22023
30		1-SYBR	methylation	21C	0.41884	0.00000	0.00000	0.00000	0.00000	10.91621	3.12917
31		1-SYBR	methylation	48C	0.74579	0.02965	0.02965	0.02096	0.02096	0.74579	0.13554
32		1-SYBR	methylation	48N	0.37413	0.11039	0.11039	0.07805	0.07805	4.85193	1.47638
33		1-SYBR	methylation	71C	0.02466	0.00974	0.00974	0.00689	0.00689	1.56886	0.61945
34		1-SYBR	methylation	82C	1.00000	0.10629	0.10629	0.06137	0.06137	1.56857	0.34870
35		1-SYBR	methylation	82N	0.30739	0.02044	0.02044	0.01445	0.01445	1.48191	0.71723
36		1-SYBR	unmethylation	208C	0.44917	0.03543	0.03543	0.02045	0.02045	5.20359	0.41041
37		1-SYBR	unmethylation	208N	0.73485	0.02894	0.02894	0.02046	0.02046	16.05092	3.49833
38		1-SYBR	unmethylation	218C	0.41204	0.06083	0.06083	0.04302	0.04302	5.89834	1.09532
39		1-SYBR	unmethylation	218N	1.00000	0.09435	0.09435	0.06671	0.06671	7.28475	0.68733
40		1-SYBR	unmethylation	21A	0.38754	0.04237	0.04237	0.02446	0.02446	5.52259	1.40537
41		1-SYBR	unmethylation	21C	0.12134	0.00419	0.00419	0.00242	0.00242	3.16258	0.91311
42		1-SYBR	unmethylation	48C	0.22735	0.00495	0.00495	0.00286	0.00286	0.22735	0.04062
43		1-SYBR	unmethylation	48N	0.95623	0.06372	0.06372	0.03679	0.03679	12.40111	1.23866
44		1-SYBR	unmethylation	82C	0.96043	0.05214	0.05214	0.03010	0.03010	1.50650	0.30529
45		1-SYBR	unmethylation	82N	0.42932	0.04053	0.04053	0.02340	0.02340	2.06969	1.01127
46		1-SYBR	methylation	28C	0.84807	0.26072	0.26072	0.18436	0.18436	4.05795	1.24754
47		1-SYBR	methylation	28N	0.68776	0.00338	0.00338	0.00239	0.00239	0.80117	0.48569
48		1-SYBR	methylation	49A	0.92656	0.02820	0.02820	0.01994	0.01994	4.60939	0.14030
49		1-SYBR	methylation	49C	0.80226	0.15761	0.15761	0.09099	0.09099	1.08001	0.28553
50		1-SYBR	methylation	71C	1.00000	0.00000	0.00000	0.00000	0.00000	2.15548	0.00000
51		1-SYBR	methylation	78C	0.29155	0.00000	0.00000	0.00000	0.00000	1.28139	0.54842
52		1-SYBR	methylation	78N	0.90556	0.45944	0.45944	0.26526	0.26526	0.90556	0.68760

	A	B	C	D	E	F	G	H	I	J	K
53		1-SYBR	methylation	8A	0.27287	0.02587	0.02587	0.01829	0.01829	19.83919	1.88088
54		1-SYBR	methylation	8C	0.77015	0.07037	0.07037	0.04063	0.04063	3.26932	0.29871
55		1-SYBR	unmethylation	28C	0.01027	0.00044	0.00044	0.00025	0.00025	0.04916	0.00209
56		1-SYBR	unmethylation	28N	0.00052	0.00187	0.00187	0.00108	0.00108	0.00061	0.00222
57		1-SYBR	unmethylation	49A	0.34288	0.04807	0.04807	0.02775	0.02775	1.70571	0.23912
58		1-SYBR	unmethylation	49C	0.49224	0.05994	0.05994	0.03461	0.03461	0.66266	0.14233
59		1-SYBR	unmethylation	78C	0.00486	0.00087	0.00087	0.00050	0.00050	0.02137	0.00992
60		1-SYBR	unmethylation	78N	1.00000	0.07356	0.07356	0.04247	0.04247	1.00000	0.56970
61		1-SYBR	unmethylation	8A	0.00545	0.00124	0.00124	0.00072	0.00072	0.39606	0.09040
62		1-SYBR	unmethylation	8C	0.00572	0.00333	0.00333	0.00192	0.00192	0.02430	0.01412
63		1-SYBR	methylation	26A	0.33013	0.04947	0.04947	0.02856	0.02856	4.29496	0.70558
64		1-SYBR	methylation	26C	0.61525	0.03815	0.03815	0.02697	0.02697	7.13873	0.44262
65		1-SYBR	methylation	54C	0.72495	0.14985	0.14985	0.08652	0.08652	1.39823	0.75513
66		1-SYBR	methylation	54N	0.66909	0.19711	0.19711	0.11380	0.11380	0.66909	0.28684
67		1-SYBR	methylation	57C	0.75624	0.21597	0.21597	0.12469	0.12469	6.60664	2.54497
68		1-SYBR	methylation	57N	0.41145	0.20332	0.20332	0.11739	0.11739	1.17717	0.62412
69		1-SYBR	methylation	59C	0.47556	0.09097	0.09097	0.05252	0.05252	7.45356	1.42574
70		1-SYBR	methylation	59N	0.29253	0.00884	0.00884	0.00625	0.00625	5.74414	1.30451
71		1-SYBR	methylation	62C	0.09485	0.04754	0.04754	0.02745	0.02745	1.11506	0.67898
72		1-SYBR	methylation	62N	0.06075	0.00229	0.00229	0.00162	0.00162	0.91784	0.23570
73		1-SYBR	methylation	71C	1.00000	0.00000	0.00000	0.00000	0.00000	6.34316	0.00000
74		1-SYBR	unmethylation	26A	0.01399	0.00212	0.00212	0.00123	0.00123	0.18201	0.03024
75		1-SYBR	unmethylation	26C	0.01049	0.00294	0.00294	0.00170	0.00170	0.12174	0.03413
76		1-SYBR	unmethylation	54C	0.57370	0.06919	0.06919	0.03995	0.03995	1.10652	0.56799
77		1-SYBR	unmethylation	54N	0.27850	0.06074	0.06074	0.03507	0.03507	0.27850	0.10589
78		1-SYBR	unmethylation	57C	0.12062	0.00107	0.00107	0.00062	0.00062	1.05380	0.27259
79		1-SYBR	unmethylation	57N	1.00000	0.07801	0.07801	0.04504	0.04504	2.86104	0.59325
80		1-SYBR	unmethylation	59C	0.67703	0.12116	0.12116	0.06995	0.06995	10.61133	1.89906
81		1-SYBR	unmethylation	59N	0.30339	0.03442	0.03442	0.01987	0.01987	5.95740	1.50161
82		1-SYBR	unmethylation	62C	0.39617	0.04660	0.04660	0.02690	0.02690	4.65725	1.70118
83		1-SYBR	unmethylation	62N	0.15787	0.02314	0.02314	0.01336	0.01336	2.38499	0.69950
84		1-SYBR	methylation	24A	0.75846	0.24996	0.24996	0.17675	0.17675	5.61994	2.27228
85		1-SYBR	methylation	24C	0.92490	0.31165	0.31165	0.17993	0.17993	2.16846	1.00621
86		1-SYBR	methylation	28A	0.49535	0.23491	0.23491	0.13563	0.13563	0.49535	0.24034
87		1-SYBR	methylation	28C	0.77130	0.14105	0.14105	0.09974	0.09974	21.01718	3.88409
88		1-SYBR	methylation	29A	1.00000	0.02753	0.02753	0.01947	0.01947	27.88093	0.76764
89		1-SYBR	methylation	29C	0.18433	0.01619	0.01619	0.01145	0.01145	4.28023	0.37589
90		1-SYBR	methylation	56C	0.56722	0.26936	0.26936	0.15551	0.15551	1.10361	0.57009
91		1-SYBR	methylation	56N	0.67925	0.23290	0.23290	0.16469	0.16469	19.01674	8.09211
92		1-SYBR	methylation	71C	0.73162	0.00000	0.00000	0.00000	0.00000	6.47836	0.00000
93		1-SYBR	unmethylation	24A	0.27362	0.05224	0.05224	0.03016	0.03016	2.02742	0.61265
94		1-SYBR	unmethylation	24C	0.10019	0.03270	0.03270	0.01888	0.01888	0.23490	0.10721
95		1-SYBR	unmethylation	28A	1.00000	0.03039	0.03039	0.01754	0.01754	1.00000	0.10696
96		1-SYBR	unmethylation	28C	0.04037	0.00339	0.00339	0.00240	0.00240	1.10009	0.09687
97		1-SYBR	unmethylation	29A	0.04493	0.01348	0.01348	0.00778	0.00778	1.25283	0.37575
98		1-SYBR	unmethylation	29C	0.28107	0.02500	0.02500	0.01443	0.01443	6.52654	0.58041
99		1-SYBR	unmethylation	56C	0.19318	0.05800	0.05800	0.03349	0.03349	0.37585	0.13629
100		1-SYBR	unmethylation	56N	0.17173	0.01718	0.01718	0.01215	0.01215	4.80780	1.30352
101		1-SYBR	methylation	23A	0.61628	0.02280	0.02280	0.01612	0.01612	9.27683	0.93270
102		1-SYBR	methylation	23C	0.26298	0.00000	0.00000	0.00000	0.00000	3.47265	0.73998
103		1-SYBR	methylation	27C	0.35139	0.00783	0.00783	0.00554	0.00554	0.84308	0.15617
104		1-SYBR	methylation	27N	0.64756	0.10016	0.10016	0.05783	0.05783	0.64756	0.12791
105		1-SYBR	methylation	3A	0.31857	0.07964	0.07964	0.04598	0.04598	1.38403	0.34600
106		1-SYBR	methylation	3C	0.72749	0.10079	0.10079	0.07127	0.07127	2.26686	0.73649

	A	B	C	D	E	F	G	H	I	J	K
107		1-SYBR	methylation	50C	0.41010	0.12210	0.12210	0.07050	0.07050	0.83715	0.24925
108		1-SYBR	methylation	50N	0.79869	0.30935	0.30935	0.21874	0.21874	4.01033	1.60624
109		1-SYBR	methylation	68C	0.28758	0.07749	0.07749	0.04474	0.04474	1.56308	0.42119
110		1-SYBR	methylation	68N	0.27752	0.05272	0.05272	0.03728	0.03728	0.33340	0.06849
111		1-SYBR	methylation	71C	1.00000	0.34785	0.34785	0.24597	0.24597	14.18405	4.93392
112		1-SYBR	unmethylation	23A	0.04626	0.00574	0.00574	0.00331	0.00331	0.69641	0.10819
113		1-SYBR	unmethylation	23C	0.46014	0.04229	0.04229	0.02442	0.02442	6.07618	1.41006
114		1-SYBR	unmethylation	27C	0.21881	0.04607	0.04607	0.02660	0.02660	0.52499	0.14676
115		1-SYBR	unmethylation	27N	0.36368	0.01908	0.01908	0.01101	0.01101	0.36368	0.04858
116		1-SYBR	unmethylation	3A	0.68575	0.09646	0.09646	0.05569	0.05569	2.97926	0.41907
117		1-SYBR	unmethylation	3C	0.55157	0.07284	0.07284	0.04206	0.04206	1.71869	0.55374
118		1-SYBR	unmethylation	50C	1.00000	0.07293	0.07293	0.04211	0.04211	2.04135	0.14889
119		1-SYBR	unmethylation	50N	0.72433	0.03744	0.03744	0.02161	0.02161	3.63694	0.41591
120		1-SYBR	unmethylation	68C	0.28128	0.00925	0.00925	0.00534	0.00534	1.52884	0.05038
121		1-SYBR	unmethylation	68N	0.76549	0.05931	0.05931	0.04194	0.04194	0.91962	0.10119
122		1-SYBR	methylation	36C	0.44624	0.01948	0.01948	0.01378	0.01378	14.77090	2.61042
123		1-SYBR	methylation	36N	0.58396	0.04010	0.04010	0.02835	0.02835	24.05095	3.27624
124		1-SYBR	methylation	44A	1.00000	0.04181	0.04181	0.02956	0.02956	1.00000	0.21093
125		1-SYBR	methylation	44C	0.32627	0.07401	0.07401	0.05233	0.05233	0.53400	0.12210
126		1-SYBR	methylation	51A	0.96551	0.15197	0.15197	0.10746	0.10746	15.75660	3.06931
127		1-SYBR	methylation	51Q	0.94099	0.26520	0.26520	0.18752	0.18752	7.13841	2.41441
128		1-SYBR	methylation	65C	0.96689	0.29734	0.29734	0.21025	0.21025	21.05964	12.54260
129		1-SYBR	methylation	65N	0.76589	0.00368	0.00368	0.00261	0.00261	19.31998	6.56982
130		1-SYBR	unmethylation	36C	1.00000	0.04842	0.04842	0.02796	0.02796	33.10066	5.89073
131		1-SYBR	unmethylation	36N	0.30393	0.03857	0.03857	0.02227	0.02227	12.51763	2.16628
132		1-SYBR	unmethylation	44A	0.26543	0.01493	0.01493	0.00862	0.00862	0.26543	0.05687
133		1-SYBR	unmethylation	44C	0.18374	0.01525	0.01525	0.00881	0.00881	0.30072	0.02641
134		1-SYBR	unmethylation	51A	0.19879	0.05011	0.05011	0.02893	0.02893	3.24409	0.89848
135		1-SYBR	unmethylation	51Q	0.51378	0.03222	0.03222	0.01860	0.01860	3.89754	0.76875
136		1-SYBR	unmethylation	65C	0.35190	0.01494	0.01494	0.01056	0.01056	7.66474	3.92281
137		1-SYBR	unmethylation	65N	0.10368	0.00888	0.00888	0.00512	0.00512	2.61550	0.91707

	L	M	N	O	P	Q	R	S	T	U	V	W
1												
2	Corrected Unscaled Expression SD	Unscaled Expression SEM	Corrected Unscaled Expression SEM	Expression	Expression SD	Corrected Expression SD	Expression SEM	Corrected Expression SEM	Wells	Mean Cq	Cq SD	Cq SEM
3	2.24876	1.59011	1.59011	16.63273	2.24876	2.24876	1.59011	1.59011	2	36.23	0.19505	0.13792
4	0.00095	0.00055	0.00055	0.00366	0.00095	0.00095	0.00055	0.00055	3	33.58	0.33565	0.19379
5	0.22276	0.15752	0.15752	1.92858	0.22276	0.22276	0.15752	0.15752	2	34.02	0.02378	0.01682
6	0.33387	0.19276	0.19276	1.00000	0.33387	0.33387	0.19276	0.19276	3	24.54	0.44799	0.25865
7	0.00211	0.00122	0.00122	0.00263	0.00211	0.00211	0.00122	0.00122	3	33.54	0.78672	0.45421
8	0.00095	0.00055	0.00055	0.00366	0.00095	0.00095	0.00055	0.00055	3	33.58	0.33565	0.19379
9	5.81640	4.11282	4.11282	18.76637	5.81640	5.81640	4.11282	4.11282	2	35.71	0.44715	0.31618
10	0.00000	0.00000	0.00000	0.32044	0.00000	0.00000	0.00000	0.00000	1	45.60	0.00000	0.00000
11	0.87671	0.50617	0.50617	1.10673	0.87671	0.87671	0.50617	0.50617	3	25.53	0.15709	0.09070
12	0.00211	0.00122	0.00122	0.00263	0.00211	0.00211	0.00122	0.00122	3	33.54	0.78672	0.45421
13	0.20564	0.11872	0.11872	0.12595	0.20564	0.20564	0.11872	0.11872	3	40.07	2.35552	1.35996
14	0.00000	0.00000	0.00000	0.32044	0.00000	0.00000	0.00000	0.00000	1	45.60	0.00000	0.00000
15	0.03178	0.01835	0.01835	0.05854	0.03178	0.03178	0.01835	0.01835	3	38.12	0.76293	0.44048
16	0.29247	0.16886	0.16886	0.26861	0.29247	0.29247	0.16886	0.16886	3	37.06	1.08910	0.62879
17	2.24876	1.59011	1.59011	16.63273	2.24876	2.24876	1.59011	1.59011	2	36.23	0.19505	0.13792
18	0.16713	0.09649	0.09649	0.15125	0.16713	0.16713	0.09649	0.09649	3	37.33	1.32617	0.76566
19	0.56982	0.32898	0.32898	2.25608	0.56982	0.56982	0.32898	0.32898	3	26.42	0.36438	0.21037
20	0.39471	0.22788	0.22788	0.26372	0.39471	0.39471	0.22788	0.22788	3	36.39	2.15925	1.24665
21	0.33387	0.19276	0.19276	1.00000	0.33387	0.33387	0.19276	0.19276	3	24.54	0.44799	0.25865
22	0.87671	0.50617	0.50617	1.10673	0.87671	0.87671	0.50617	0.50617	3	25.53	0.15709	0.09070
23	0.75643	0.43672	0.43672	10.15168	0.75643	0.75643	0.43672	0.43672	3	27.46	0.10750	0.06206
24	0.60324	0.34828	0.34828	0.86984	0.60324	0.60324	0.34828	0.34828	3	25.32	0.46743	0.26987
25	0.39486	0.27921	0.27921	7.50335	0.39486	0.39486	0.27921	0.27921	2	34.31	0.07592	0.05368
26	1.67017	1.18099	1.18099	6.95616	1.67017	1.67017	1.18099	1.18099	2	35.34	0.15602	0.11032
27	1.50565	0.86928	0.86928	9.98282	1.50565	1.50565	0.86928	0.86928	3	34.21	0.14471	0.08355
28	2.02421	1.43133	1.43133	5.11976	2.02421	2.02421	1.43133	1.43133	2	34.20	0.57040	0.40333
29	0.22023	0.15572	0.15572	0.57670	0.22023	0.22023	0.15572	0.15572	2	38.31	0.44001	0.31113
30	3.12917	3.12917	3.12917	10.91621	3.12917	3.12917	3.12917	3.12917	1	34.94	0.00000	0.00000
31	0.13554	0.09584	0.09584	0.74579	0.13554	0.13554	0.09584	0.09584	2	34.11	0.05735	0.04055
32	1.47638	1.04396	1.04396	4.85193	1.47638	1.47638	1.04396	1.04396	2	35.11	0.42566	0.30099
33	0.61945	0.43802	0.43802	1.56886	0.61945	0.61945	0.43802	0.43802	2	39.03	0.56964	0.40279
34	0.34870	0.20132	0.20132	1.56857	0.34870	0.34870	0.20132	0.20132	3	33.69	0.15335	0.08854
35	0.71723	0.50716	0.50716	1.48191	0.71723	0.71723	0.50716	0.50716	2	35.39	0.09593	0.06783
36	0.41041	0.23695	0.23695	5.20359	0.41041	0.41041	0.23695	0.23695	3	26.32	0.11379	0.06569
37	3.49833	2.47369	2.47369	16.05092	3.49833	3.49833	2.47369	2.47369	2	25.61	0.05681	0.04017
38	1.09532	0.77451	0.77451	5.89834	1.09532	1.09532	0.77451	0.77451	2	26.45	0.21300	0.15061
39	0.68733	0.48602	0.48602	7.28475	0.68733	0.68733	0.48602	0.48602	2	25.17	0.13612	0.09625
40	1.40537	0.81139	0.81139	5.52259	1.40537	1.40537	0.81139	0.81139	3	26.53	0.15772	0.09106
41	0.91311	0.52718	0.52718	3.16258	0.91311	0.91311	0.52718	0.52718	3	28.21	0.04977	0.02874
42	0.04062	0.02345	0.02345	0.22735	0.04062	0.04062	0.02345	0.02345	3	27.30	0.03141	0.01813
43	1.23866	0.71514	0.71514	12.40111	1.23866	1.23866	0.71514	0.71514	3	25.23	0.09613	0.05550
44	0.30529	0.17626	0.17626	1.50650	0.30529	0.30529	0.17626	0.17626	3	25.22	0.07832	0.04522
45	1.01127	0.58386	0.58386	2.06969	1.01127	1.01127	0.58386	0.58386	3	26.39	0.13621	0.07864
46	1.24754	0.88214	0.88214	4.05795	1.24754	1.24754	0.88214	0.88214	2	33.70	0.44353	0.31362
47	0.48569	0.34343	0.34343	0.80117	0.48569	0.48569	0.34343	0.34343	2	34.00	0.00710	0.00502
48	0.14030	0.09921	0.09921	4.60939	0.14030	0.14030	0.09921	0.09921	2	33.57	0.04391	0.03105
49	0.28553	0.16485	0.16485	1.08001	0.28553	0.28553	0.16485	0.16485	3	33.78	0.28342	0.16363
50	0.00000	0.00000	0.00000	2.15548	0.00000	0.00000	0.00000	0.00000	1	33.46	0.00000	0.00000
51	0.54842	0.54842	0.54842	1.28139	0.54842	0.54842	0.54842	0.54842	1	35.24	0.00000	0.00000
52	0.68760	0.39699	0.39699	0.90556	0.68760	0.68760	0.39699	0.39699	3	33.60	0.73196	0.42260

	L	M	N	O	P	Q	R	S	T	U	V	W
53	1.88088	1.32998	1.32998	19.83919	1.88088	1.88088	1.32998	1.32998	2	35.33	0.13678	0.09672
54	0.29871	0.17246	0.17246	3.26932	0.29871	0.29871	0.17246	0.17246	3	33.84	0.13182	0.07610
55	0.00209	0.00121	0.00121	0.04916	0.00209	0.00209	0.00121	0.00121	3	31.11	0.06130	0.03539
56	0.00222	0.00128	0.00128	0.00061	0.00222	0.00222	0.00128	0.00128	3	35.41	5.16450	2.98172
57	0.23912	0.13805	0.13805	1.70571	0.23912	0.23912	0.13805	0.13805	3	26.05	0.20225	0.11677
58	0.14233	0.08217	0.08217	0.66266	0.14233	0.14233	0.08217	0.08217	3	25.53	0.17569	0.10143
59	0.00992	0.00573	0.00573	0.02137	0.00992	0.00992	0.00573	0.00573	3	32.19	0.25892	0.14949
60	0.56970	0.32892	0.32892	1.00000	0.56970	0.56970	0.32892	0.32892	3	24.51	0.10613	0.06127
61	0.09040	0.05219	0.05219	0.39606	0.09040	0.09040	0.05219	0.05219	3	32.03	0.32930	0.19012
62	0.01412	0.00815	0.00815	0.02430	0.01412	0.01412	0.00815	0.00815	3	31.96	0.83817	0.48392
63	0.70558	0.40737	0.40737	4.29496	0.70558	0.70558	0.40737	0.40737	3	32.92	0.21617	0.12481
64	0.44262	0.31298	0.31298	7.13873	0.44262	0.44262	0.31298	0.31298	2	32.03	0.08945	0.06325
65	0.75513	0.43597	0.43597	1.39823	0.75513	0.75513	0.43597	0.43597	3	31.79	0.29821	0.17217
66	0.28684	0.16561	0.16561	0.66909	0.28684	0.28684	0.16561	0.16561	3	31.91	0.42502	0.24538
67	2.54497	1.46934	1.46934	6.60664	2.54497	2.54497	1.46934	1.46934	3	31.73	0.41201	0.23787
68	0.62412	0.36034	0.36034	1.17717	0.62412	0.62412	0.36034	0.36034	3	32.61	0.71292	0.41160
69	1.42574	0.82315	0.82315	7.45356	1.42574	1.42574	0.82315	0.82315	3	32.40	0.27596	0.15933
70	1.30451	0.92243	0.92243	5.74414	1.30451	1.30451	0.92243	0.92243	2	33.10	0.04360	0.03083
71	0.67898	0.39201	0.39201	1.11506	0.67898	0.67898	0.39201	0.39201	3	34.72	0.72305	0.41745
72	0.23570	0.16667	0.16667	0.91784	0.23570	0.23570	0.16667	0.16667	2	35.37	0.05429	0.03839
73	0.00000	0.00000	0.00000	6.34316	0.00000	0.00000	0.00000	0.00000	1	31.33	0.00000	0.00000
74	0.03024	0.01746	0.01746	0.18201	0.03024	0.03024	0.01746	0.01746	3	30.51	0.21908	0.12648
75	0.03413	0.01970	0.01970	0.12174	0.03413	0.03413	0.01970	0.01970	3	30.92	0.40439	0.23348
76	0.56799	0.32793	0.32793	1.10652	0.56799	0.56799	0.32793	0.32793	3	25.15	0.17400	0.10046
77	0.10589	0.06114	0.06114	0.27850	0.10589	0.10589	0.06114	0.06114	3	26.19	0.31467	0.18168
78	0.27259	0.15738	0.15738	1.05380	0.27259	0.27259	0.15738	0.15738	3	27.40	0.01277	0.00737
79	0.59325	0.34251	0.34251	2.86104	0.59325	0.59325	0.34251	0.34251	3	24.35	0.11255	0.06498
80	1.89906	1.09642	1.09642	10.61133	1.89906	1.89906	1.09642	1.09642	3	24.91	0.25819	0.14907
81	1.50161	0.86696	0.86696	5.95740	1.50161	1.50161	0.86696	0.86696	3	26.07	0.16367	0.09450
82	1.70118	0.98218	0.98218	4.65725	1.70118	1.70118	0.98218	0.98218	3	25.68	0.16970	0.09798
83	0.69950	0.40385	0.40385	2.38499	0.69950	0.69950	0.40385	0.40385	3	27.01	0.21148	0.12210
84	2.27228	1.60675	1.60675	5.61994	2.27228	2.27228	1.60675	1.60675	2	32.29	0.47546	0.33620
85	1.00621	0.58093	0.58093	2.16846	1.00621	1.00621	0.58093	0.58093	3	32.00	0.48612	0.28066
86	0.24034	0.13876	0.13876	0.49535	0.24034	0.24034	0.13876	0.13876	3	32.91	0.68418	0.39501
87	3.88409	2.74647	2.74647	21.01718	3.88409	3.88409	2.74647	2.74647	2	32.27	0.26384	0.18656
88	0.76764	0.54280	0.54280	27.88093	0.76764	0.76764	0.54280	0.54280	2	31.89	0.03972	0.02809
89	0.37589	0.26580	0.26580	4.28023	0.37589	0.37589	0.26580	0.26580	2	34.33	0.12670	0.08959
90	0.57009	0.32914	0.32914	1.10361	0.57009	0.57009	0.32914	0.32914	3	32.71	0.68510	0.39554
91	8.09211	5.72198	5.72198	19.01674	8.09211	8.09211	5.72198	5.72198	2	32.45	0.49467	0.34979
92	0.00000	0.00000	0.00000	6.47836	0.00000	0.00000	0.00000	0.00000	1	32.34	0.00000	0.00000
93	0.61265	0.35371	0.35371	2.02742	0.61265	0.61265	0.35371	0.35371	3	25.39	0.27543	0.15902
94	0.10721	0.06190	0.06190	0.23490	0.10721	0.10721	0.06190	0.06190	3	26.84	0.47085	0.27185
95	0.10696	0.06175	0.06175	1.00000	0.10696	0.10696	0.06175	0.06175	3	23.52	0.04384	0.02531
96	0.09687	0.06850	0.06850	1.10009	0.09687	0.09687	0.06850	0.06850	2	28.15	0.12109	0.08562
97	0.37575	0.21694	0.21694	1.25283	0.37575	0.37575	0.21694	0.21694	3	27.99	0.43270	0.24982
98	0.58041	0.33510	0.33510	6.52654	0.58041	0.58041	0.33510	0.33510	3	25.35	0.12830	0.07407
99	0.13629	0.07868	0.07868	0.37585	0.13629	0.13629	0.07868	0.07868	3	25.89	0.43317	0.25009
100	1.30352	0.92172	0.92172	4.80780	1.30352	1.30352	0.92172	0.92172	2	26.06	0.14430	0.10204
101	0.93270	0.65952	0.65952	9.27683	0.93270	0.93270	0.65952	0.65952	2	34.48	0.05337	0.03774
102	0.73998	0.73998	0.73998	3.47265	0.73998	0.73998	0.73998	0.73998	1	35.71	0.00000	0.00000
103	0.15617	0.11043	0.11043	0.84308	0.15617	0.15617	0.11043	0.11043	2	35.29	0.03214	0.02273
104	0.12791	0.07385	0.07385	0.64756	0.12791	0.12791	0.07385	0.07385	3	34.41	0.22315	0.12883
105	0.34600	0.19977	0.19977	1.38403	0.34600	0.34600	0.19977	0.19977	3	35.43	0.36067	0.20823
106	0.73649	0.52078	0.52078	2.26686	0.73649	0.73649	0.52078	0.52078	2	34.24	0.19988	0.14134

	L	M	N	O	P	Q	R	S	T	U	V	W
107	0.24925	0.14391	0.14391	0.83715	0.24925	0.24925	0.14391	0.14391	3	35.07	0.42955	0.24800
108	1.60624	1.13579	1.13579	4.01033	1.60624	1.60624	1.13579	1.13579	2	34.11	0.55878	0.39512
109	0.42119	0.24318	0.24318	1.56308	0.42119	0.42119	0.24318	0.24318	3	35.58	0.38874	0.22444
110	0.06849	0.04843	0.04843	0.33340	0.06849	0.06849	0.04843	0.04843	2	35.63	0.27409	0.19381
111	4.93392	3.48881	3.48881	14.18405	4.93392	4.93392	3.48881	3.48881	2	33.78	0.50184	0.35486
112	0.10819	0.06246	0.06246	0.69641	0.10819	0.10819	0.06246	0.06246	3	28.91	0.17900	0.10334
113	1.41006	0.81410	0.81410	6.07618	1.41006	1.41006	0.81410	0.81410	3	25.60	0.13259	0.07655
114	0.14676	0.08473	0.08473	0.52499	0.14676	0.14676	0.08473	0.08473	3	26.67	0.30377	0.17538
115	0.04858	0.02805	0.02805	0.36368	0.04858	0.04858	0.02805	0.02805	3	25.94	0.07568	0.04369
116	0.41907	0.24195	0.24195	2.97926	0.41907	0.41907	0.24195	0.24195	3	25.02	0.20293	0.11716
117	0.55374	0.31970	0.31970	1.71869	0.55374	0.55374	0.31970	0.31970	3	25.34	0.19053	0.11000
118	0.14889	0.08596	0.08596	2.04135	0.14889	0.14889	0.08596	0.08596	3	24.48	0.10522	0.06075
119	0.41591	0.24013	0.24013	3.63694	0.41591	0.41591	0.24013	0.24013	3	24.94	0.07456	0.04305
120	0.05038	0.02909	0.02909	1.52884	0.05038	0.05038	0.02909	0.02909	3	26.31	0.04746	0.02740
121	0.10119	0.07155	0.07155	0.91962	0.10119	0.10119	0.07155	0.07155	2	24.86	0.11179	0.07905
122	2.61042	1.84585	1.84585	14.77090	2.61042	2.61042	1.84585	1.84585	2	36.32	0.06298	0.04454
123	3.27624	2.31665	2.31665	24.05095	3.27624	3.27624	2.31665	2.31665	2	35.93	0.09906	0.07005
124	0.21093	0.14915	0.14915	1.00000	0.21093	0.21093	0.14915	0.14915	2	35.16	0.06031	0.04265
125	0.12210	0.08634	0.08634	0.53400	0.12210	0.12210	0.08634	0.08634	2	36.77	0.32727	0.23141
126	3.06931	2.17033	2.17033	15.75660	3.06931	3.06931	2.17033	2.17033	2	35.21	0.22708	0.16057
127	2.41441	1.70725	1.70725	7.13841	2.41441	2.41441	1.70725	1.70725	2	35.24	0.40659	0.28750
128	12.54260	8.86896	8.86896	21.05964	12.54260	12.54260	8.86896	8.86896	2	35.20	0.44367	0.31372
129	6.56982	4.64556	4.64556	19.31998	6.56982	6.56982	4.64556	4.64556	2	35.54	0.00694	0.00491
130	5.89073	3.40102	3.40102	33.10066	5.89073	5.89073	3.40102	3.40102	3	24.25	0.06986	0.04033
131	2.16628	1.25070	1.25070	12.51763	2.16628	2.16628	1.25070	1.25070	3	25.96	0.18310	0.10571
132	0.05687	0.03283	0.03283	0.26543	0.05687	0.05687	0.03283	0.03283	3	26.16	0.08116	0.04686
133	0.02641	0.01525	0.01525	0.30072	0.02641	0.02641	0.01525	0.01525	3	26.69	0.11977	0.06915
134	0.89848	0.51874	0.51874	3.24409	0.89848	0.89848	0.51874	0.51874	3	26.58	0.36365	0.20995
135	0.76875	0.44384	0.44384	3.89754	0.76875	0.76875	0.44384	0.44384	3	25.21	0.09046	0.05223
136	3.92281	2.77385	2.77385	7.66474	3.92281	3.92281	2.77385	2.77385	2	25.75	0.06124	0.04330
137	0.91707	0.52947	0.52947	2.61550	0.91707	0.91707	0.52947	0.52947	3	27.52	0.12350	0.07130
